# Different needs in patients with schizophrenia spectrum disorders who behave aggressively towards others depend on gender: a latent class analysis approach

**DOI:** 10.1186/s12991-021-00343-5

**Published:** 2021-03-13

**Authors:** Moritz Philipp Günther, Steffen Lau, Sabine Kling, Martina Sonnweber, Elmar Habermeyer, Johannes Kirchebner

**Affiliations:** 1 Department of Consultation-Liaison-Psychiatry and Psychosomatic Medicine, University Hospital Zurich, Zurich, Switzerland; 2 Department of Forensic Psychiatry, University Hospital of Psychiatry Zurich, Zurich, Switzerland; 3 Computer Vision Laboratory, Department of Information Technology and Electrical Engineering, Swiss Federal Institute of Technology (ETH) Zurich, Zurich, Switzerland

**Keywords:** Women, Men, Gender differences, Forensic psychiatry, Offenders with schizophrenia spectrum disorder, Latent class analysis, Female, Male

## Abstract

**Background:**

There is limited research with inconsistent findings on differences between female and male offender patients with a schizophrenia spectrum disorder (SSD), who behave aggressively towards others. This study aimed to analyse inhomogeneities in the dataset and to explore, if gender can account for those.

**Methods:**

Latent class analysis was used to analyse a mixed forensic dataset consisting of 31 female and 329 male offender patients with SSD, who were accused or convicted of a criminal offence and were admitted to forensic psychiatric inpatient treatment between 1982 and 2016 in Switzerland.

**Results:**

Two homogenous subgroups were identified among SSD symptoms and offence characteristics in forensic SSD patients that can be attributed to gender. Despite an overall less severe criminal and medical history, the female-dominated class was more likely to receive longer prison terms, similarly high antipsychotic dosages, and was less likely to benefit from inpatient treatment. Earlier findings were confirmed and extended in terms of socio-demographic variables, diseases and criminal history, comorbidities (including substance use), the types of offences committed in the past and as index offence, accountability assumed in court, punishment adjudicated, antipsychotic treatment received, and the development of symptoms during psychiatric inpatient treatment.

**Conclusions:**

Female offender patients with schizophrenia might need a more tailored approach in prevention, assessment and treatment to diminish tendencies of inequity shown in this study.

## Background

An increase in the number of females entering forensic mental health care and the penitentiary system has been observed in many countries (for reviews, see [1–3]. Gender differences were found in both criminal behaviour and various aspects of schizophrenia. The present study aims to explore without (even statistical) preconceptions if gender may explain relevant differences in the histories and treatment needs of patients with schizophrenia spectrum disorder (SSD) who underwent forensic psychiatric inpatient treatment.

Psychiatric research found gender-based differences regarding onset and course of SSD [4–6]: Women were found to become affected 4–6 years later than men, to experience more comorbidities, but less alcohol and illicit drug use, to benefit from better socio-economic circumstances (e.g. being in a relationship, employed) and to better respond to treatment, often requiring lower antipsychotic dosages.

Criminological research has also identified a gender gap [7–16]: women display less violence and aggressive behaviour overall and engage in less serious crimes. However, some studies have argued that female aggressive behaviour is not as overt as similar behaviour in males and is, therefore, less likely to lead to prosecution, which may lead to inaccurate estimates of female violence [12]. Others suggested that the gender gap in violence is largely due to males being more prone to neurocognitive deficits, difficult temperament and hyperactivity paired with poor parenting skills [11], excessive androgen production, thyroid dysfunction, Cushing’s syndrome and congenital adrenal hyperplasia [17].

Research on individuals with severe mental illness (SMI) in particular evidenced higher rates of violent behaviour (odds ratio (OR) 2.49–6.6 for men; OR 14.9–23.2 for women), any convictions (OR 2.15–3.4 for men; OR 2.85–3.7 for women) and also victimisation (87% lifetime prevalence for both men and women) in comparison to the general population [18–23]. In addition, substance use disorders were found to be a major risk factor for violence in individuals with SMI [24–26]. Similarly, conduct disorder prior to age 15 and antisocial personality disorder during adulthood were identified as major risk factors, even after controlling for alcohol and illicit drug use [27–30].

Offending women with SMI were found to start committing crimes at a later average age (24.9 years) than men (20.8 years), receive less severe punishment or shorter prison terms (18.4 months for women, 23.4 months for men) or were more likely to be considered to have diminished or no responsibility due to mental illness (28% women, 12% men; [31]. As for the type of crime, a study comparing male and female homicide offenders with SMI reported a four to one ratio of males to females [16]. In contradiction, other studies found that women with SMI committed more serious crimes than men, more frequently including arson (27.5% women, 12.4% men) and homicide (28.1% women, 15.1% men; [31], and were primarily admitted for such crimes [32, 33]. Female offenders with SMI were found to engage in more self-harming (women 22–33%, men 8–13%) and to have comorbid personality disorders [34]. Several studies found female offender patients with SMI to be more likely to target close family members [16, 31], which has not been found in female offenders without SMI. In contrast to non-offending women with SSD [5, 6], many female offender patients with SMI did abuse alcohol (34–48%) or illicit drugs (35–44%), suffered from socio-economic adversity [32, 33] and had a higher number of forensic and general psychiatric hospitalizations than men [34]. The few studies explicitly focusing on female offender patients with SMI [31–34] did not distinguish between different mental disorders and often presented descriptive accounts only. While SSD was a frequent diagnosis in these studies, only one study in Hunan Province, China, (to our knowledge) explored female (homicide) offenders with SSD [16]. Results may not generalise to western societies, but indicated a 4:1 male to female ratio of homicide with males being more influenced by delusions (46% vs. 35%) and females more frequently targeting close family members (62% vs. 41%).

Overall, there is a need for further research on differences between specific subgroups of patients with SSD who have committed criminal offences. For this purpose, variables similar to those in the reviewed findings, augmented by detail in their categorization, are to be analysed using latent class analysis (LCA). LCA is a statistical approach specifically designed for the identification of inherent unobservable (i.e. latent) classes within a particular dataset. Another objective is to explore the understudied topic of treatment outcome in offender patients with SSD [35] Results should allow for new insights into criminal behaviour of forensic patients with SSD and offer implications for increased efficacy in treatment and risk management.

## Methodology

### Source and primary processing of data

The study was approved by the Zurich Cantonal Ethics Committee (Ref.-No. KEK-ZH-NR 2014-0480). Medical files of all 370 offender patients with a schizophrenia spectrum disorder (31 female, 339 male), as defined in chapters 295.0 to 295.9 of the 9th revision of the international classification of diseases (ICD-9) [36] and chapters F20.0 to F25.9 of the 10th revision of the international statistical classification of diseases (ICD-10) [37], who were admitted to the Centre for Inpatient Forensic Therapies at the Zurich University Hospital of Psychiatry between 1982 and 2016, were analysed retrospectively. No files were excluded. As an institution run by the Zurich health authorities, the centre provides treatment for both men and women, who have committed a crime that is related to a mental disorder and for whom an expert opinion has concluded that psychiatric treatment can reduce the risk of future crimes. Files were reviewed with regard to criminal and medical histories, psychiatric inpatient and outpatient reports, police reports, court proceedings (including testimonies), reports from social workers, and biannual reports from physicians and nursing staff during forensic inpatient treatment. The composition and categorization of the final set of 63 variables for quantitative analysis was informed by prior research reviewed in the introduction and can be found in Table 2. For conversion of cumulative antipsychotic dosages into olanzapine equivalents, the classical weighted mean dose method [38] was employed. If older antipsychotics were prescribed, the minimum effective dose method [39] or international experts’ consensus based olanzapine equivalents [40] provided the necessary converting factors. Changes in psychopathology over forensic inpatient treatment were assessed using the cumulative difference between positive, negative and general psychopathology between admission and discharge.

A close adoption of the Positive and Negative Symptom Scale (PANSS) was used to categorise and quantify psychopathological symptoms (30 subcategories; symptom being fully present, somewhat present, or absent) during content analysis [41].

Retrospective file analysis by means of directed qualitative content analysis [42] used a standardised questionnaire and rating protocol [43, 44] adopted from a set of criteria first established by Seifert [45]. A trained independent physician systematically reviewed all case files and a second similarly trained independent rater encoded a random subsample of 10% of cases assuring inter-rater reliability, Cohen’s Kappa [46] being 0.78.

### Data analysis

#### Background on latent class analysis (LCA)

Supervised statistical techniques have to be distinguished from unsupervised techniques.

Supervised methods, such as linear/logistic regressions, trees, supported vector machines, naïve Bayes and other, define an outcome a priori (e.g. male/female). They also define possible predictors (e.g. violent behaviour yes/no) for that outcome and explore their significance. This means, it is crucial to define a hypothesis beforehand, which is mostly derived from existing literature or past observations (e.g. men show more violent behaviour than women). The supervised mathematical model is calculated to asses if the predictor variable can significantly distinguish between outcomes and if this corresponds to the hypothesis (e.g. aggressive behaviour does significantly distinguish between men/women and men show significantly more of it).

By contrast, unsupervised methods, such as principle component analysis, cluster analysis and LCA (LCA, even though more progressive, has many similarities to cluster analysis in its way of identifying classes instead of clusters) do not require the definition of a hypothesis in advance (e.g. there are differences due to gender). This means there are no statistical prejudices before data analysis (LCA rather explores how many homogenous subgroups the dataset is composed of). All possible variables (e.g. aggressive behaviour, age of illness onset) are entered into the mathematical modelling process. Then homogenous groups/clusters/classes are extracted so that the homogeneity within a class is maximised and inter-class differences are also maximised. The result can be only one group, meaning the variables are not helpful in distinguishing classes. The result can also be two classes or more, meaning the variables are indeed helpful in defining different classes. For each class, the LCA model calculates class conditional item response probabilities—describing the probability of how often a given variable category is represented within a class. After this step, one can further explore, which external variable might best explain why the groups identified in LCA differ from each other. E.g. based on hypotheses, we can select a specific covariate (e.g. gender) and verify if the same classes are identified. If the same set of classes can be explained by the covariate (similar to a regression analysis for this particular number of classes), this is providing evidence that this variable (e.g. gender) is indeed distinguishing groups.

In contrast to supervised methods, unsupervised methods such as LCA have hardly any assumptions a priori and are, therefore, mainly used for explorative research. Thus, they impose less prejudice on data analysis.

#### Specific technical procedures used

**Fig. 1:**
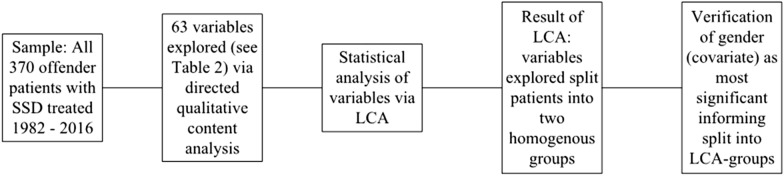
Flow chart of methodology. Note. SSD = schizophrenia spectrum disorder; LCA = latent class analysis

The specifics of data analysis are summarised in Fig. 1.

R Studio version 1.1.383 was used in conjunction with the poLCA package for latent class analysis (LCA). LCA is a type of finite mixture model designed for the analysis of multivariate categorical (instead of just dichotomous or continuous) data grouping all observations into unobserved (= latent) homogenous nominal classes by probability, while minimising confounding between observations.

To find the most parsimonious (i.e. balancing the goodness of fit with the number of model parameters involved) model representing the entire dataset of 63 items and 370 observations, solutions with one, two, and three classes were evaluated. [Solutions with more than three classes were not evaluated because results on the three class solution indicated, that a smaller number of classes (i.e. two classes) would be more parsimonious (see Table 1).] Based on presentations in extant literature employing LCAs [47, 48], the following criteria were estimated to evaluate model fit: maximum log-likelihood, log-likelihood Chi-square (G^2^) statistics, Bayesian Information Criterion (BIC), Akaike Information Criterion (AIC) and entropy. Each of these criteria has different strengths and weaknesses in assessing validity and reliability of the final model. While BIC and AIC are parsimony measures aiming to avoid overfitting, maximum log-likelihood and G^2^ are measures of goodness of model fit only. Entropy is a measure of classification uncertainty, with values of > 0.8 being suggested for a good separation between classes [49]. AIC and BIC have both been used as criteria to select the optimal number of (latent) classes in the past [50]. BIC measures the trade-off between model fit and complexity of the model and penalises additional model parameters stronger than AIC, which is why it can be considered to be more conservative by preventing that a better model fit is achieved by simply increasing model complexity. Since AIC may overestimate the correct number of components in a finite mixture model [51], BIC is more suitable in selecting the best fitting model [50]. The sample-size-corrected BIC (scBIC) is a value computed for completeness. For better comparison with previous literature, different model evaluation criteria are reported even though BIC is given highest priority.

**Table 1:** Summary of different LCA model fit criteria

Number of classes	Number of estimated parameters	Residual degrees of freedom	Maximum log-likelihood	AIC	BIC	scBIC	Entropy	Number of times solution was found
1 (without covariate)	96	274	− 11195	22582	22957	23221	–	500/500
**2** (with covariate)	193	177	− 10677	2741	**22496**	23024	0.8806	491/500
**2** (without covariate)	194	176	− 1674	21737	**22496**	23027	0.8827	488/500
3 (without covariate)	290	80	− 10475	21530	22665	23460	0.889	32/500

BIC is considered the most relevant criterion for model selection according to which the two-class model indicated best model fit (highlighted with bold type). In the two-class model with covariate, initialisation of the priors was based on the potential class-predictor *gender*, but did not show any relevant differences to the two-class model without covariate. For the purpose of this study, subsequent results were based on the two-class model with covariate. Higher values of maximum log-likelihood indicate a better model fit but favour overfitting. Information criteria penalise the number of estimated parameters to prevent overfitting: AIC, Akaike’s Information Criterion; BIC, Bayesian Information Criterion; scBIC, sample-size-corrected Bayesian Information Criterion; Lower AIC, BIC and scBIC values indicate a good and parsimonious model fit. Entropy, measure of classification uncertainty with higher numbers indicating a better class separation; number of times solution was found = number of times solution was found out of 500 random initializations of prior probabilities to avoid local extrema, with higher numbers indicating a more unambiguous result

For a given number of classes, the aim of the statistical process was to find the best fitting model by maximising the log-likelihood function via an expectation maximisation (EM) algorithm. The process was repeated 500 times for each number of classes evaluated with different starting values to avoid local extrema.

To investigate the hypothesis that the identified classes are a function of gender, a latent class regression model was fitted to the dataset in addition to the basic latent class model described above. The regression model was implemented by including a covariate (i.e. gender) in the LCA analysis, which accounts for a potential predictor variable of class membership. In contrast to the basic latent class model, where each patient has the same prior probability of class membership, in the latent class regression model, the prior probability of belonging to a particular class is allowed to vary based on the covariate [52]. The last step, exploring a latent class regression model based on the covariate gender, was used to confirm that the two-class solution is best explained by gender. It is inherent to this methodology, that the number of male and female subjects responsible for variable observations do not need to be balanced.

## Results

**Fig. 2:**
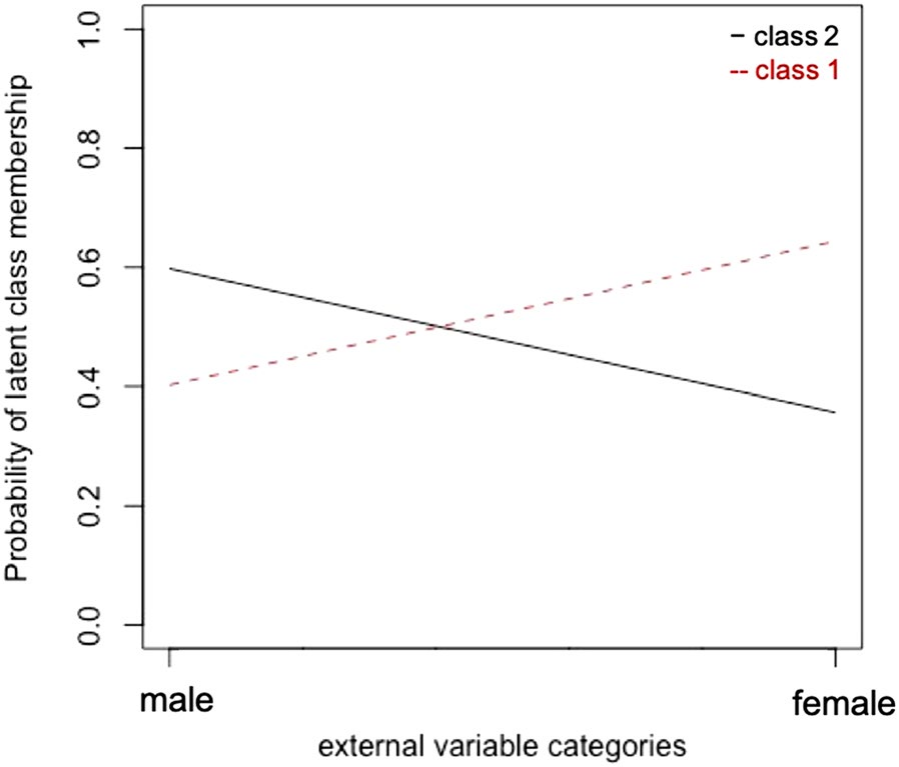
Differentiability of two distinct offender subgroups. Note. X-axis: subgroups suggested by LCA with covariate (female/ male gender); Y-axis: probability of subgroup membership based on manifestations of all variables explored on a scale from 0 to 1. Dashed line represents class 1, continuous line class 2

Based on the criteria discussed above, the two-class-model (with and without covariate) was identified to represent the most parsimonious model fit as measured by the lowest BIC value among the tested models (Fig. 2, Table 1). The two-class-model with gender as a covariate had the same parsimonious model fit (BIC value), thus indicating no relevant difference between both two-class models. This means, the model without covariate evidenced that two separate classes are identifiable. The model with covariate confirmed that gender is an adequate predictor of class membership. Figure 2 visualises the probability of male and female offender patients in the two identified groups. It shows that gender can account for some differences between the two identified classes. LCA identified the two groups based on all specified variables. Subsequent results and discussion are based on the two-class model with gender as a covariate.

LCA provided the item response probabilities of their categories for a given variable and class. All results are presented in Table 2. Differences in probability of a given category between the two classes of above 10% are considered clinically most relevant, as previously done in similar research [53], and have been set in bold type in Table 2.

**Table 2:** Class conditional item response probabilities of the two classes (i.e. male and female dominated)

All variables explored in present study	Interclass differences in item response probability	Class 1 (women)	Class 2 (men)
Relationship status at index offence			
Single	**0.2475**	0.5222	0.7697
Married	0.0986	0.1887	0.0901
Married, living separated	0.0315	0.0703	0.0388
Divorced	0.0875	0.1795	0.092
Widowed	0.0281	0.0328	0.0047
In a committed relationship	0.0019	0.0066	0.0047
Homelessness at time of index offence			
No	**0.1447**	0.8837	0.739
Yes	**0.1447**	0.1163	0.261
Highest level of education completed successfully			
No graduation	0	0	0
Primary school	0.004	0.2579	0.2619
Secondary school (high school)	**0.2991**	0.1769	0.476
Baccalaureate	0.0763	0.127	0.0507
Vocational school	0	0	0
College	**0.1408**	0.3464	0.2056
University	0	0	0
Unknown	0.086	0.0918	0.0058
Number of criminal registry entries			
0	0	0	0
1	**0.2892**	0.4612	0.172
2–3	0.014	0.2691	0.2831
4–8	0.0844	0.1671	0.2515
> 8	**0.1908**	0.1026	0.2934
Number of previous criminal convictions			
0	**0.2005**	0.6952	0.4947
1–2	0.0559	0.1208	0.1767
> 2	**0.1446**	0.184	0.3286
Age at first criminal registry entry			
< 21	**0.3154**	0.1122	0.4276
21–35	0.0784	0.3947	0.4731
> 35	**0.3939**	0.4931	0.0992
Previous offences: homicide/attempted homicide			
No	0.0342	0.9512	0.9854
Yes	0.0342	0.0488	0.0146
Previous offences: assault			
No	0.0984	0.6811	0.5827
Yes	0.0984	0.3189	0.4173
Previous offences: threat, coercion			
No	0.0894	0.7738	0.6844
Yes	0.0894	0.2262	0.3156
Previous offences: sexual abuse of children			
No	0.0249	0.9677	0.9926
Yes	0.0249	0.0323	0.0074
Previous offences: rape, sexual assault			
No	0.0279	0.9556	0.9835
Yes	0.0279	0.0444	0.0165
Previous offences: other sexual offence			
No	0.02	0.951	0.971
Yes	0.02	0.049	0.029
Previous offences: property crime without violence			
No	**0.2271**	0.755	0.5279
Yes	**0.2271**	0.245	0.4721
Previous offences: property crime with violence			
No	**0.1909**	0.9802	0.7893
Yes	**0.1909**	0.0198	0.2107
Previous offences: arson			
No	0.0115	0.9315	0.943
Yes	0.0115	0.0685	0.057
Previous offences: criminal damage			
No	**0.1672**	0.8292	0.662
Yes	**0.1672**	0.1708	0.338
Previous offences: traffic offence			
No	**0.1874**	0.9085	0.7211
Yes	**0.1874**	0.0915	0.2789
Previous offences: controlled substances act			
No	**0.4873**	0.9048	0.4175
Yes	**0.4873**	0.0952	0.5825
Previous offences: weapons act			
No	0.0931	0.9733	0.8802
Yes	0.0931	0.0267	0.1198
Index offence: homicide/attempted homicide			
No	**0.1748**	0.6085	0.7833
Yes	**0.1748**	0.3915	0.2167
Index offence: assault			
No	0.0999	0.6527	0.5528
Yes	0.0999	0.3473	0.4472
Index offence: threat, coercion			
No	0.0516	0.7363	0.6847
Yes	0.0516	0.2637	0.3153
Index offence: sexual abuse of children			
No	0.0093	0.9809	0.9716
Yes	0.0093	0.0191	0.0284
Index offence: rape, sexual assault			
No	0.0755	0.962	0.8865
Yes	0.0755	0.038	0.1135
Index offence: other sexual offence			
No	0.0585	0.9658	0.9073
Yes	0.0585	0.0342	0.0927
Index offence: property crime without violence			
No	**0.1512**	0.9078	0.7566
Yes	**0.1512**	0.0922	0.2434
Index offence: property crime with violence			
No	0.0669	0.9788	0.9119
Yes	0.0669	0.0212	0.0881
Index offence: arson			
No	0.0386	0.9217	0.8831
Yes	0.0386	0.0783	0.1169
Index offence: criminal damage			
No	0.0523	0.8861	0.8338
Yes	0.0523	0.1139	0.1662
Index offence: traffic offence			
No	0.0298	0.9601	0.9303
Yes	0.0298	0.0399	0.0697
Index offence: controlled substances act			
No	**0.2138**	0.9641	0.7503
Yes	**0.2138**	0.0359	0.2497
Index offence: weapons act			
No	0.0192	0.9567	0.9375
Yes	0.0192	0.0433	0.0625
Index offence: misuse of emergency system			
No	0.0172	0.9936	0.9764
Yes	0.0172	0.0064	0.0236
Victim(s) of index offence had close relationship to patient			
No	**0.1096**	0.7964	0.906
Yes	**0.1096**	0.2036	0.094
Victims of index offence were the patient’s parents			
No	0.0362	0.9522	0.916
Yes	0.0362	0.0478	0.084
Victims of index offence were the patient’s siblings			
No	0.0337	0.9899	0.9562
Yes	0.0337	0.0101	0.0438
Victims of index offence were the patient’s offspring			
No	0.0482	0.9461	0.9943
Yes	0.0482	0.0539	0.0057
Victims of index offence were other relatives of the patient			
No	0.0114	1	0.9886
Yes	0.0114	0	0.0114
Victim of index offence had any form of relationship to patient			
Yes, some form of relationship	**0.11**	0.72	0.61
None	**0.11**	0.28	0.39
Victim of abuse by a related person			
No	**0.1112**	0.8435	0.9547
Yes	**0.1112**	0.1565	0.0453
Victim of any crime			
No	0.0247	0.852	0.8767
Yes	0.0247	0.148	0.1233
Criminal responsibility as per judgement			
Yes, fully	0.049	0.3215	0.3705
Yes, partially	0.0353	0.1144	0.1497
None	0.0298	0.4865	0.4567
No judgement	0.046	0.0691	0.0231
Unknown	0.0086	0.0086	0
Prison term in months for index offence			
≤ 13	0.0282	0.3511	0.3229
14–48	**0.2589**	0.2054	0.4643
> 48	**0.2307**	0.4435	0.2128
Age at first diagnosis of schizophrenia			
< 21	**0.2422**	0.1198	0.362
21–35	0.001	0.5307	0.5317
> 35	**0.2432**	0.3495	0.1063
Age at first psychiatric inpatient treatment			
< 21	**0.2951**	0.1452	0.4403
21–35	0.0432	0.5137	0.4705
> 35	**0.252**	0.3411	0.0891
Number of psychiatric inpatient treatments			
≤ 1	**0.1691**	0.5207	0.3516
2–4	0.0029	0.2615	0.2586
> 4	**0.1721**	0.2178	0.3899
Alcohol use at any time			
No use	**0.3996**	0.6246	0.225
Abuse	**0.2692**	0.2414	0.5106
Misuse	**0.1304**	0.134	0.2644
Any illegal substance use at any time			
Yes	**0.624**	0.3666	0.9906
No	**0.624**	0.6334	0.0094
Cannabis use at any time			
No	**0.7485**	0.8296	0.0811
Yes	**0.7485**	0.1704	0.9189
Opioid use at any time			
No	**0.4677**	0.9917	0.524
Yes	**0.4677**	0.0083	0.476
Cocaine use at any time			
No	**0.5522**	0.9865	0.4343
Yes	**0.5522**	0.0135	0.5657
Amphetamine, ecstasy or other illegal stimulant use at any time			
No	**0.3837**	1	0.6163
Yes	**0.3837**	0	0.3837
Self-injurious behaviour at any time			
No	0.0796	0.5809	0.5013
Yes	0.0796	0.4191	0.4987
Attempted suicide at any time			
No	0.0356	0.6877	0.6521
Yes	0.0356	0.3123	0.3479
Suicidal ideation during forensic inpatient treatment			
No	0	0.7966	0.7966
Yes	0	0.2034	0.2034
Personality disorder diagnosed prior to admission			
Yes	0.0635	0.0909	0.1544
No	0.0635	0.9091	0.8456
Psychiatric or somatic comorbidity			
No	0.0607	0.6927	0.632
Yes	0.0607	0.3073	0.368
Total time spent in prison			
0	0.0917	0.2273	0.1356
≤ 4 weeks	0.06	0.179	0.119
≤ 12 months	0.0726	0.3138	0.3864
1–2 years	0.0617	0.07	0.1317
> 2 years	0.0174	0.2099	0.2273
Total time spent in forensic inpatient treatment			
1–11 weeks	**0.1757**	0.4376	0.2619
12–150 weeks	**0.1232**	0.2606	0.3838
> 150 weeks	0.0525	0.3018	0.3543
Olanzapine equivalent dose at discharge from forensic inpatient treatment			
≤ 25,7 mg	0.0313	0.7519	0.7206
> 25,7 mg	0.0313	0.2481	0.2794
Change in positive symptoms over forensic inpatient treatment			
Worsening	0	0	0
Unchanged	**0.1131**	0.5212	0.4081
Slightly better	0.0944	0.4448	0.5392
Substantially better	0.0187	0.034	0.0527
Change in negative symptoms over forensic inpatient treatment			
Worsening	0.0172	0.0068	0.024
Unchanged	**0.1282**	0.6817	0.5535
Slightly better	**0.1097**	0.2842	0.3939
Substantially better	0.0014	0.0273	0.0287
Change in overall psychopathology over forensic inpatient treatment			
Worsening	0.0048	0	0.0048
Unchanged	**0.128**	0.6366	0.5086
Slightly better	**0.1252**	0.3566	0.4818
Substantially better	0.002	0.0068	0.0048

Interclass differences in item response probability above 10% are in bold type

Present findings indicated that compared to the male-dominated class, offender patients in the female-dominated class were less likely to be single and have experienced homelessness, but more likely to have a higher level of formal education. The female-dominated class was more likely to have fewer criminal registry entries, fewer criminal convictions, and started to commit crimes at an older age. For previous offences, the female-dominated class was less likely to commit property crimes, criminal damage, traffic offences, or offences against the narcotics act. For index offences, it was somewhat more likely to attempt or commit homicide, but less likely to commit non-violent property crimes, or offences against the narcotics act. Victims of the female-dominated class were more likely to have had a close relationship to the offender patients. The female-dominated class was more likely to be themselves victims of sexual traumatisation inflicted by a related person, but not of any other type of crime. This class was similarly likely to be judged responsible for their offence, but more likely to receive a longer sentence for their index offence than the male-dominated class.

Results on medical histories showed the female-dominated class to be diagnosed with SSD and be admitted to their first psychiatric inpatient treatment at an older age. This class was more likely to have fewer psychiatric inpatient treatments, less likely to misuse or abuse alcohol and much less likely to use illegal substances. Male- and female-dominated classes showed no relevant difference in terms of self-injurious behaviour, attempted suicide, suicidal ideation during treatment, or the diagnosis of a personality disorder. The female-dominated class was less likely to undergo long-term forensic psychiatric treatment, but equally likely to receive high doses of antipsychotics. Women were less likely to benefit from forensic inpatient treatment regarding remission of psychopathological symptoms of SSD.

## Discussion

Results confirmed a tendency towards inequality between female and male offender patients suffering from SSD and provided new details using more narrowly defined variables instead of broad categories used in existing literature. Prior research provided inconsistent results on whether women were held insufficiently [31] or excessively [33] accountable for their offending and the amount of punishment (prison term) received. Present results indicated the female-dominated class was similarly likely to be judged accountable for their offence as the male-dominated class, but more likely to receive a longer prison sentence than men. The latter may be influenced by the higher probability of the female-dominated class to attempt or commit homicide. The female-dominated class also tended to more frequently target individuals to whom they had a close relationship, including their own children (5% difference in probability). This may be considered to be particularly atrocious in the legal culture the present study was set in and may have been penalised with longer prison sentences [54]. As in prior reports [31, 34], the female-dominated class seemed less likely to commit sexual offences. A higher prevalence of arson [31, 34] could not be confirmed in the female-dominated class, which may be caused by the low prevalence of female arsonists in the present sample. Similar to one study on offender patients with SMI in the Netherlands [31], but in contrast to a study set in China [16] and research on non-mentally ill offenders in the US and UK [7, 14], present results indicated a higher probability for the female-dominated class to engage in homicide or attempted homicide. Besides cultural aspects, present findings may have been skewed by not including offender patients with SSD waiting in prisons to be transferred into forensic psychiatric treatment [55, 56] and who may be more likely to have committed less serious crimes.

Previous findings reported non-offending female patients with SSD to better maintain intimate relationships [5, 6]. Yet, in our sample, the female-dominated class also seemed less likely to be single than males, but they more frequently lived in separation from their spouse or had been divorced, as has been described for offenders in general regardless of a mental disorder [57]. Maintaining relationships might be a skill needing more therapeutic attention in offending women versus non-offending women with SSD. Similar to results in reviews on gender differences among non-offending patients with SSD [5, 6, 58], female offender patients in the present study were also likely to be older at first diagnosis of SSD and first inpatient treatment, have experienced fewer psychiatric inpatient treatments, have fewer comorbidities, have been married, have a higher level of formal education and have not been homeless. Previous findings suggested that female offenders with SMI are more likely to abuse alcohol and illegal substances [32], have more prior inpatient treatments and have a higher probability of being diagnosed with a personality disorder and self-harm [34]. This could not be confirmed here and may reflect particularities in the psychiatric understanding of the role of personality traits either as chronic symptoms of SSD or as discrete comorbidity [34]. Discrepant results may also reflect the inclusion of a wide spectrum of psychiatric diagnoses in the samples explored in past research [32].

Females in our sample were less likely than men to experience remission in psychopathology over inpatient treatment. Similar gender differences have been reported for non-offending patients with SSD [5, 6]. While treatment objectives in offender patients include the prevention of further violence in addition to a remission of psychopathology [59], the latter may be an important mediator in this respect [60]. Clinicians should consider prescribing lower antipsychotic dosages, as was recommended for non-violent women with SSD due to differences in absorption and metabolism between the sexes, resulting in women being overdosed at standard doses and consequently experiencing more side effects [5, 6]. In addition, adjunctive treatment with oestrogen may yield ameliorated treatment outcomes due to its neuroprotective effects [61, 62].

Many female offenders experienced psychological, physical or sexual violence, often in connection with relationships, especially with men (relatives and partners). This is also the case with the female-dominated class in the current study (see Table 2). Recent research confirmed violent victimisation to be a better predictor of violent behaviour than current psychopathology [63]. The current and extant studies evidence that patients with SSD tend to act against those with whom they have a close relationship—oftentimes their mothers (in male offender patients) or their children (in female offender patients) [31], which may be due to unwanted childbirth [5, 33], or serious post-partum depression and psychosis [64].

Violence against family or close acquaintances in the past will pose particular challenges to inpatient treatment of offender women with SSD in comparison to non-offending women with SSD because it complicates treatment programs aiming at social reintegration [65]. For instance, family therapy sessions may be difficult or impossible after serious violence of a patient against a family member and/ or abuse through a family member—both of which occurred more frequently in the female-dominated group in the present study (see Table 2). Further, a history of violence in close relationships may render both offender female patients with SSD and the psychiatric team treating them more reluctant to form therapeutic alliances out of fear of recurrence of such violent and hurtful phenomena. Gender-specific training of staff, raising awareness for such challenges, can help to master them and significantly improve the recovery process of patients [66]. In addition, women may benefit from more trauma-specific interventions—especially since histories of abuse impeding treatment success are frequently overlooked [32, 33]. Women were found to favour being in a single-gender environment since they may feel safer and more comfortable talking to other women about their experiences [67], which should encourage forensic mental health institutions to build separate women’s wards. Since women may process violent victimisation differently than men and to overcome risk-relevant behaviour patterns, treatment in a specially protected and protecting environment is necessary. Current forensic psychiatry (in which patients of the present study were treated) may fail to recognise and respond to specific treatment needs of female patients with SSD, which might provide additional explanation as to why the female-dominated group experienced less of a remission of positive and negative psychopathology in comparison to the male-dominated group. More gender-sensitive treatment [61, 62], which requires a better understanding regarding the different needs of male and female offender patients, should be provided.

Thus, the findings of this study may be of use to researchers and institutions interested in developing a more gender-sensitive approach to female forensic patients with SSD. Future research should further explore specific treatment needs in the management of patients with SSD and evaluate the benefits of specialised treatment facilities providing highly specific treatment, often referred to as personalised medicine, which may account for other differences between offending patient subgroups beyond gender [53].

## Limitations

Limitations have already been addressed in the interpretation of results and in the “Methods”. They involve the known weaknesses of retrospective file analysis, including human error in the documentation of events, recording of events over a prolonged period of time with changing cultural aspects and treatment options, the selection and categorization of screening parameters for coding and coding itself. Further limitations involve selection effects (one forensic psychiatric institution in Switzerland) and the limited number of patients explored. In addition, the assessment of antipsychotic dosing might be biased by differences in age, weight and PANSS values among the two identified classes. Furthermore, the dataset had relatively unequal sample sizes with respect to gender (339 male vs 31 female). While similar sample size is not a prerequisite for LCA, at relatively small overall sample sizes (< 500), the associated danger is a worse detectability of the class with low prevalence [68]. The fact that the two-class solution was identified as the best fitting model, and that there was no other underlying latent parameter that could explain the difference between the two classes, however, strongly suggests that the model correctly identified gender. It might yet explain why the model did not achieve a perfect separation between male and female. Future studies balancing out these factors are needed.

## Conclusion

Capitalising on LCA without any a priori assumptions, this study provides evidence that the investigated dataset on SSD symptoms and offence characteristics of forensic patients with SSD consists of two homogenous groups and shows that these subgroups can in part be attributed to gender. Results confirmed recently summarised differences between male and female patients with SSD [5, 6] for the largely unexplored subgroup of female-dominated offender patients and addressed inconsistencies raised in a scarce body of research on this subgroup [16, 31–34]. It calls upon clinicians to help reduce any disadvantage for female offender patients with SSDs by acknowledging that women have different treatment needs from men in a number of aspects and by adopting new treatment approaches to address specific treatment needs.

## Data Availability

The datasets used and/or analysed during the current study are available from the corresponding author on reasonable request.
